# A Common Lesion of the Horse

**Published:** 1888-10

**Authors:** Daniel D. Lee

**Affiliations:** Instructor in Anatomy and Operative Veterinary Surgery, Harvard University


### Art. XXIII—A COMMON LESION OF THE HORSE.

BY DANIEL D. LEE, M. D. V.

*Instructor in Anatomy and Operative Veterinary Surgery, Harvard University.*

Under the names of "Shoe Ball," "Shoe Boil" and "Capped Elbow," is described a common lesion of the horse, and one that causes annoyance more from the blemish that it causes than for the time that it unfits the animal for work.

It is caused either by the heel of the shoe of the opposite fore foot coming against the elbow, or by the toe of

* References in the Bible to the use of the horse among the Jews: Genesis, xii., 15, 16; xiii., 2–5; xxxi., 17, 18; xxxii., 5–13–15; xli., 42, 43; xlv., 19–21, 27; xlvi., 29–32–34; xlvii., 16–17; l., 7, 8, 9. Exodus, xii., 37, 38; ix., 3, 4; xiv., 6–9, 17, 18, 23, 25, 26, 28–31; xv., 1. Moses' command to future king: Deuteronomy, xvii., 15, 16. Punishment of animals, Exodus, xxi., 28–36; theft of animals, Exodus, xxii., 1–5; wandering of animals, Exodus, xxiii., 4, 5; wandering of animals, Deuteronomy, xxii., 1–4; stolen and borrowed animals, Exodus, xxiii., 9–16; dislike of dogs, Exodus, xxiii., 30, 31; numbers of animals possessed, Numbers, xxxi., 32–35, 42–45; ham stringing enemy's horses, Joshua, xi., 4–6; ignorance of shoeing, Judges, vi., 21, 22.

the shoe of the hind foot of the same side, in both cases the animal being in the recumbent position, caulked shoes making the injury more severe. It is most frequent in coarse bred horses. Where the skin glides over the oleeranon, it is provided with a bursa mucosa which lubricates it; this becoming inflamed forms a hot, tense, painful swelling, filled with a clear or bloody serous fluid, poured out by the inflamed bursa. When this lesion occurs for the first time, if the horse be prevented from lying down and proper treatment adopted, it will generally be absorbed. If, after the swelling has appeared, the horse be allowed to lie down, the pressure of the heel of the shoe may either cause the swelling to burst and discharge, or it may inflame it to a greater extent and set up suppuration, the remaining contents and discharge becoming purulent.

Generally, after the attack, a greater or less thickening of the skin and tissues remains, this thickened portion being constantly irritated by the shoe when the animal lies down, eventually results in a tumor of dense, fibrous character, which may weigh several pounds, and is generally sessile and more or less diffuse, but at other times it, by its weight, stretches the skin and hangs down as far even as the carpus, becoming pedunculated. When one of these tumors is present it is liable at any time to get injured when the animal lies down and to be the seat of an acute inflammatory process. These tumors are especially liable to injury if the animal gets cast for a time and struggles to get up. Such attacks generally unfit the animal for work for several days. I have sometimes thought that the intensity of the lameness was too great, particularly in cases of the first attack, to be accounted for by such a superficial injury, and I have thought that the synovial bursa between the common tendon of the caput magnum and caput medium muscles and the oleeranon must have been involved as well.

*Treatment.*—In the first attack, if the swelling is not very great, perfect rest and hot fomentations, the horse at the same time being kept tied up with a strong halter. This will reduce the swelling in mild attacks if the first time. If the swelling is very great, or if it is already opened by the horse having laid with the heel of the shoe against it, I advise making a free incision in the centre of the tumor, washing it out and injecting some caustic once or twice, being very careful that no fistulous traits formed in the healing. The horse should be prevented from lying down as long as possible, a week or ten days at least.

In cases where a fibrous tumor is present I think it best not to operate until an acute attack occurs, for then the skin is stretched by the swelling and you have much more skin to hide the cicatrix after healing. It is, of course, however, necessary to operate at once if the tumor is of great size.

In these fibrous tumors are found often fistulous openings, lined by a pyrogenic membrane, and never healing entirely, but sometimes closing at their external end and retaining the secretion of the pyrogenic membrane, which then sets up inflammation without any external injury having been received from the shoe, and is a constant source of trouble till the tumor is removed. In operation for removal, first make a longitudinal incision through the centre of the tumor to the healthy tissue underneath, then dissect out each half, taking with it all diseased skin. If the tumor is diffuse and is not then all removed, slough out the rest with caustic injections. As soon as the granulating surface forms the horse may be put to work and allowed to lie down, the cavity from which the tumor was removed kept clean and occasionally stimulated with caustic. When the tumor is pedunculated remove with the ecraseur. The hæmorrhage which follows the removal of these tumors can be controlled by injecting very cold or very hot water. By very hot, I mean as hot as one can bear the hand in. If this fails the cavity may be temporarily plugged with tow and the edges sewed together, or some styptic used, the stitches taken out and the dressing removed as soon as the hæmorrhage has been controlled.

Preventative treatment is very important in these cases, as horses generally have more than one attack. It consists first of what may be done to prevent the fore foot from causing the injury—a boot must be worn which will cover the hock of the foot. These are made of canvass and leather; or a piece of scantling may be placed across the floor of the stall in such a position that the sternum will rest on it when the horse lies down. This scantling should be three by four inches. This raises the body and forearm and keeps the elbow above the foot, at the same time making it hard for the foot to be brought back under the body, and very uncomfortable if it is. This scantling should be padded or well covered with straw.

If the hind foot causes the trouble the horse should have a girth of leather or canvass made in such a way that it can be stuffed with straw so that it projects five or six inches. This should be strapped on at night just behind the fore legs. It raises the body from the floor and also prevents the hind foot from reaching the elbow.

A similar lesion to "shoe ball," and one also caused by the heel of the shoe of the fore foot, is seen, though rarely, on the keel of the sternum between the fore legs. This is also an inflamed bursa mucosa. It should always be opened, and the horse kept standing for a few days for it to heal. It seldom recurs, as the position when the foot comes violently against it is not often taken. From its location it is not a conspicuous blemish, even when acute. It is more common in cows, where it is generally due to the animal having to lie on a badly made floor or on the edge of the manger.

